# Redefining a martial art as modern leisure: the primacy of entertainment motivation in adult taekwondo

**DOI:** 10.3389/fpsyg.2026.1768627

**Published:** 2026-03-24

**Authors:** Byeongseok Min

**Affiliations:** Division of Martial Arts and Guard, University of SunMoon, Asan-si, Chungcheongnam-do, Republic of Korea

**Keywords:** continuance intention, leisure satisfaction, leisure studies, participation motivation, taekwondo

## Abstract

**Introduction:**

As a traditional martial art, Taekwondo’s modern identity as a leisure activity for adults is increasingly contested, particularly in its country of origin. This study investigates this phenomenon by examining the motivational structure that underpins adult participation.

**Methods:**

Focusing on the relationships between participation motivation, leisure satisfaction, and continuance intention, data from 233 adult practitioners in South Korea, consisting primarily of young male students, were analysed using a structural equation model (SEM).

**Results:**

The findings reveal a significant shift in leisure values. It is observed that traditional motivations such as ‘self-development’ and ‘family’ orientation were excluded during the measurement validation phase, which may indicate a reweighting of motivational structures or potential measurement adequacy issues within this specific cohort. Instead, while health and social motivations remain relevant, entertainment orientation emerges as the primary driver for both satisfaction and continued participation.

**Discussion:**

This result challenges the conventional understanding of discipline-focused activities within leisure studies, suggesting that the pursuit of fun and enjoyment is becoming a central tenet even in traditional practices. The findings suggest the possibility that prioritizing experiential and hedonic aspects may be necessary to ensure the sustained relevance of such activities among specific demographics, such as young adult practitioners.

## 1 Introduction

Taekwondo, a traditional Korean martial art, has achieved remarkable success as a global sport and an official Olympic event (Ahn et al., 2009), primarily through its competitive sparring discipline, Kyorugi. However, a paradox exists within its home country, South Korea, where its relevance as a serious leisure pursuit for adults is diminishing. The practice is increasingly perceived as a form of childcare rather than a fulfilling activity for all ages (An, 2014), a situation compounded by internal industry challenges (Lee and Na, 2014). This has led to a documented decline in public interest among adults (Choi and Yeo, 2021) and a pessimistic outlook among its future professionals (Kim and Kwon, 2021; Min, 2019).

This phenomenon offers a compelling case study for leisure scholars, illustrating the challenge traditional physical activities face in adapting to a contemporary leisure landscape. While previous leisure research has extensively explored sport participation motivation in general populations, often examining demographic differences (Ahmed et al., 2020; Ley, 2020) or its effect on life satisfaction (Guo and Yu, 2022), a significant gap exists in understanding these motivational structures within the context of traditional, discipline-based practices transitioning into modern leisure offerings. How do classic motivations for martial arts, such as self-development, compete with the modern pursuit of enjoyment and entertainment? This question is critical for understanding evolving leisure choices in societies where diverse options compete for individuals’ limited free time.

This study addresses this gap by empirically investigating the causal relationships between the participation motivations of adult Taekwondo practitioners and key experiential outcomes: leisure satisfaction and continuance intention. By identifying the most influential drivers, this research moves beyond a purely managerial focus on retention. Instead, it aims to contribute to a deeper theoretical understanding of how a traditional martial art is being redefined as a modern leisure activity, offering insights into the shifting values that shape contemporary leisure engagement.

Motivation is the fundamental driver of human behaviour, initiating and sustaining goal-oriented actions. In the context of sports and leisure, Deci and Ryan's (1985) Self-Determination Theory (SDT) is a foundational framework. SDT posits that fulfilling three innate psychological needs—autonomy, competence, and relatedness—fosters intrinsic motivation, enhancing engagement in an activity (Calvo et al., 2010). Building on this, various motivation scales have been developed to understand why individuals engage in leisure. A prominent model adapted for the Korean context categorises leisure motivations into factors such as health, social, and entertainment orientations. These motivations represent the specific benefits individuals seek from their leisure activities. While Self-Determination Theory (SDT) provides a foundational framework for understanding the diverse psychological needs driving leisure, this study utilizes it strictly as a theoretical backdrop to categorize motivation sub-factors rather than directly testing its psychological mechanisms. Furthermore, rather than directly measuring the theoretical constructs of the Experience Economy, this study indirectly explores the quality of the leisure experience by examining the predictive power of entertainment-oriented motivations.

To understand the shifting motivational landscape in activities like Taekwondo, it is useful to employ the Serious Leisure Perspective (SLP), a cornerstone of contemporary leisure theory (Stebbins, 1992). Stebbins (1992) categorises leisure into two main types: ‘serious’ and ‘casual’. Serious leisure requires significant personal effort, knowledge, and training, often leading to long-term fulfilment and a sense of identity (e.g., amateur musicians, dedicated hobbyists). Traditionally, martial arts like Taekwondo, with their structured progression and emphasis on discipline, epitomise the qualities of serious leisure.

This trend is further explained by the concept of the Experience Economy, which argues that consumers increasingly seek memorable experiences rather than mere services (Pine and Gilmore, 1999). In a leisure context, this means a Taekwondo dojang is no longer just a provider of an instructional ‘service’ but a facilitator of a holistic ‘experience’. According to this framework, the value lies not in the techniques taught, but in the overall enjoyment and engagement felt during the activity. This shifts the provider’s focus from technical delivery to staging an environment that is enjoyable, immersive, and emotionally engaging. Therefore, motivations tied directly to the quality of the immediate experience, such as entertainment, are likely to become more influential in determining participant satisfaction and loyalty than motivations tied to the transactional delivery of a skill.

The preceding theoretical discussion suggests that while various motivations drive participation, those aligned with the principles of modern leisure consumption—namely, the pursuit of satisfying experiences—will have the most significant impact on behavioural outcomes. When participation in a sport fulfills an individual’s motivations, their sense of satisfaction naturally increases (Beard and Ragheb, 1980). Within the context of the Experience Economy, motivations centered on the quality of the experience itself (i.e., entertainment) are logically expected to have the strongest link to satisfaction. Previous studies within Taekwondo have confirmed that higher motivation is linked to greater leisure satisfaction (Koh and Ko, 2014; Yoo et al., 2010). Therefore, we propose:

*H1*: Sports participation motivation is positively related to leisure satisfaction.

Similarly, motivation acts as the initial impetus for the intention to continue an activity. In a competitive leisure market, the experiences that provide the most potent and immediate rewards are most likely to foster persistence (Huta and Ryan, 2010). Thus, motivations that are more hedonic and experiential in nature should strongly predict an individual’s desire to continue. This link is supported by research showing that higher motivation among Taekwondo trainees leads to a greater intention to continue their training (Hwang, 2020; Jang, 2021). Therefore, we propose:

*H2*: Sports participation motivation is positively related to continuous exercise intention.

Finally, the relationship between satisfaction and loyalty is well-established in consumer and leisure literature. A satisfying leisure experience, as defined within the Experience Economy, is a powerful trigger for the decision to participate again (Pine and Gilmore, 1999). This causal link has been demonstrated across various leisure contexts (Ahn and Shin, 2021; Loncaric et al., 2018). Thus, we hypothesize:

*H3*: Leisure satisfaction is positively related to continuous exercise intention.

## 2 Materials and methods

### 2.1 Participants and procedure

The target population for this study comprised adult Taekwondo practitioners in South Korea. To test the hypotheses, a non-probability convenience sampling method was employed. A structured questionnaire was distributed to adult men and women at Taekwondo centres in Seoul and Gyeonggi-do, Korea, over a 20-day period from March 1 to March 20, 2022. Although formal Institutional Review Board (IRB) approval was exempted for this study due to its observational nature and the absence of sensitive data collection, strict ethical guidelines were consistently observed. Prior to participation, informed consent was obtained from all individuals. The survey was completely voluntary, and it was explicitly stated that no personally identifiable information would be collected, thereby ensuring complete anonymity throughout the research process. After removing 17 incomplete or unfaithful responses from an initial sample of 250, a total of 233 valid questionnaires were used for the final analysis. The sample was predominantly male (81.9%), in their 20s (85.2%), and students (86.9%). While this demographic profile presents clear limitations for generalisability to all adult practitioners, it is crucial to note that this skew accurately reflects the current consumer landscape of adult Taekwondo in Korea, which is heavily concentrated in university clubs and dojangs catering to this specific young-adult demographic (Min, 2019; Kim and Kwon, 2021). Therefore, this study provides focused and externally valid insights into the dominant and most active segment of the adult participant market, rather than a generalized adult population.

### 2.2 Measurement

All measurement items were adapted from established scales and assessed on a 5-point Likert-type scale, ranging from 1 (‘strongly disagree’) to 5 (‘strongly agree’). Sports Participation Motivation: This was measured using the scale developed by Kim and Lee (1995), which adapts the work of Iso-Ahola and Allen (1982) specifically for the Korean leisure context. The scale originally comprised five factors: family, health, self-development, social, and entertainment orientations. These factors were selected to deliberately test the relative importance of traditional martial art values (self-development, family) against contemporary leisure values (health, social, entertainment). This scale was chosen for its proven applicability to leisure activities in an East Asian cultural setting. Leisure Satisfaction: This was assessed using a four-item scale adapted from Lim et al. (2011), based on the foundational work of Beard and Ragheb (1980). This scale is widely recognised in leisure studies for its reliability in capturing the affective and cognitive fulfillment derived from a leisure experience. Continuous Exercise Intention: A three-item scale was developed for this study based on established behavioural intention literature. The items (‘I will continue Taekwondo,’ ‘I will talk about the positive aspects of Taekwondo to those around me,’ and ‘I will do Taekwondo for the rest of my life if conditions permit’) were designed to capture the multifaceted nature of loyalty, encompassing both personal commitment and advocacy.

### 2.3 Data analysis

The collected data were analysed using SPSS 26.0 and AMOS 26.0. The analysis followed the recommended two-step approach for structural equation modelling (SEM). First, a confirmatory factor analysis (CFA) was conducted to assess the measurement model’s reliability and validity. We evaluated composite reliability (CR), average variance extracted (AVE), and discriminant validity. Second, the structural model was tested to examine the hypothesised paths. The model’s goodness-of-fit was evaluated using multiple indices, including the Comparative Fit Index (CFI), the Tucker-Lewis Index (TLI), and the Root Mean Square Error of Approximation (RMSEA), with established thresholds (e.g., CFI/TLI > 0.90, RMSEA <0.08) used to determine an acceptable fit. Statistical significance was set at *p* < 0.05.

## 3 Results

### 3.1 Measurement model

Prior to testing the hypotheses, a confirmatory factor analysis (CFA) was conducted to assess the validity and reliability of the measurement model. An initial CFA indicated that two factors from the original sports participation motivation scale—family orientation and self-development orientation—had non-significant factor loadings and were thus removed. This outcome is a critical finding in itself, moving beyond a simple procedural step. It provides the first empirical evidence that traditional martial art values (self-development, family) do not hold significant motivational currency for this modern cohort. The implications of this finding are discussed in detail in the discussion section. After removing these factors, the revised measurement model, consisting of the remaining three motivation factors and the two outcome variables, demonstrated a satisfactory fit to the data: S-B *χ*^2^ = 236.570, df = 91, *p* < 0.001, CFI = 0.943, TLI = 0.923, RMSEA = 0.090. As detailed in Table 1, all constructs showed strong internal consistency (Cronbach’s *α* ranging from 0.79 to 0.90). Convergent validity was established, with Average Variance Extracted (AVE) values (ranging from 0.62 to 0.73) exceeding the recommended 0.50 threshold. Discriminant validity was also confirmed, as the square root of each construct’s AVE was greater than its correlations with other constructs (see Table 2). These results confirmed that the measurement scales were reliable and valid, permitting a confident progression to the structural model analysis.

**Table 1:** Measurement properties of scale items.

Items	CR	λ	*α*	AVE
*Health orientation*	0.83		0.83	0.62
To relieve physical and mental tension		0.850		
To refresh myself		0.772		
To maintain and improve physical strength and health		0.730		
*Social orientation*	0.81		0.79	0.68
To build a harmonious relationship between friends and colleagues		0.943		
To make friends with neighbors by exercising		0.687		
*Entertainment orientation*	0.79		0.79	0.66
It’s fun to work out		0.831		
To feel refreshed after exercising		0.792		
*Leisure satisfaction*	0.90		0.90	0.69
I became socially healthy through Taekwondo.		0.894		
I became physically healthy through Taekwondo.		0.807		
I became mentally healthy through Taekwondo.		0.829		
I am generally satisfied with Taekwondo as an aspect of my leisure.		0.794		
*Continuous exercise intention*	0.89		0.88	0.73
I will continue Taekwondo.		0.932		
I will talk about the positive aspects of Taekwondo around me.		0.862		
I will do Taekwondo for the rest of my life if conditions permit me.		0.766		

**Table 2:** Means, standard deviations, correlations, and discriminant validity estimates.

	Health orientation	Social orientation	Entertainment orientation	Leisure satisfaction	Continuous exercise intention
(1) Health orientation	*0.787*				
(2) Social orientation	0.658**	*0.825*			
(3) Entertainment orientation	0.589**	0.295**	*0.812*		
(4) Leisure satisfaction	0.514**	0.260**	0.772**	*0.831*	
(5) Continuous exercise intention	0.297**	0.130*	0.611**	0.587**	*0.854*
Mean	3.466	3.494	4.150	4.089	4.391
SD	1.060	0.992	0.908	0.901	0.756

The values listed on each diagonal are the square root AVE; *p < 0.05 (two-tailed); **p < 0.01 (two-tailed).

### 3.2 Structural model assessment and hypotheses testing

**Figure 1:**
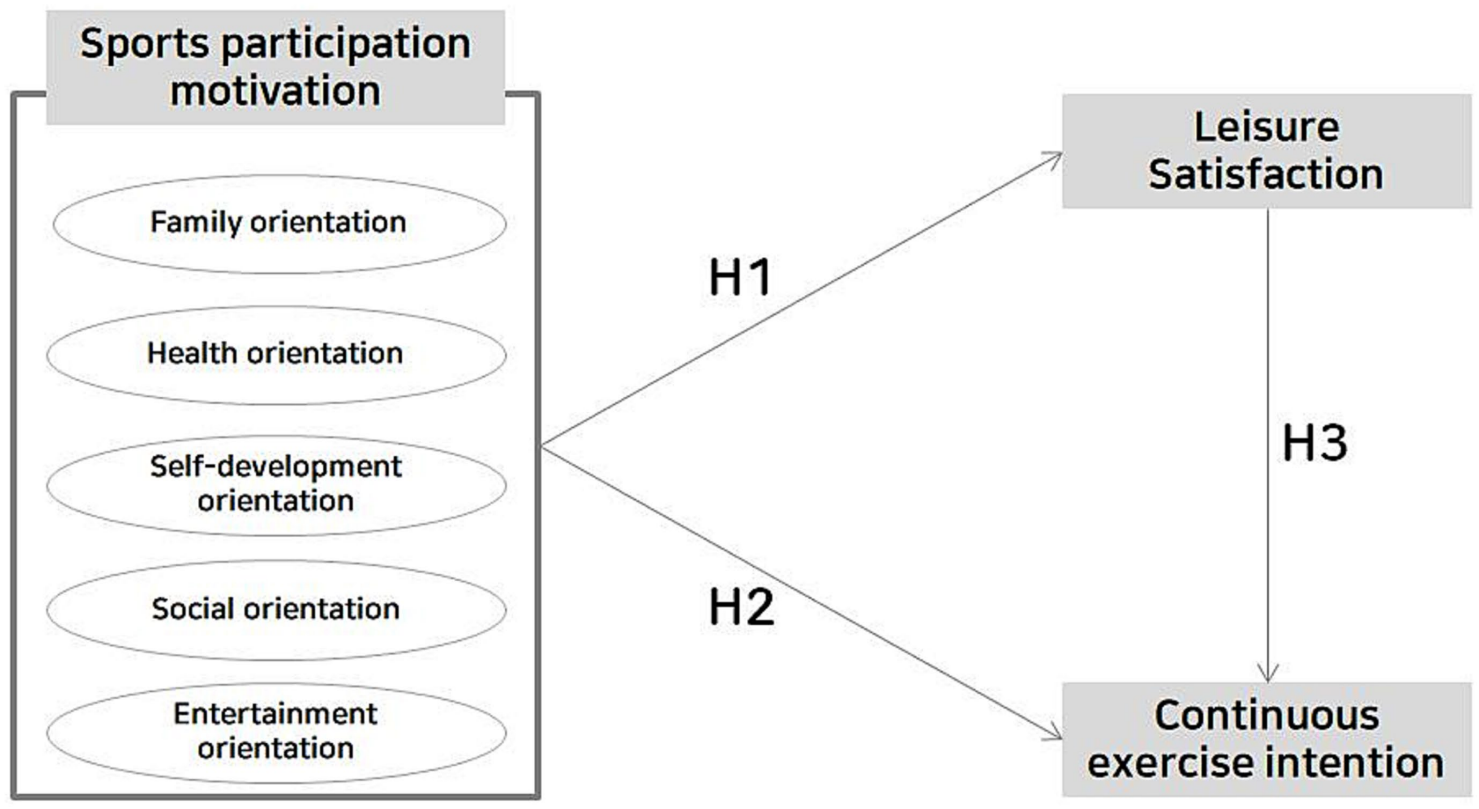
Conceptual framework and research hypotheses of the study.

Following the validation of the measurement model, the hypothesised structural model was tested to examine the relationships between the latent variables. The structural model also demonstrated an acceptable fit to the data (CFI = 0.918, TLI = 0.909, RMSEA = 0.098). Although the RMSEA values of 0.090 for the measurement model and 0.098 for the structural model slightly exceed the stringent 0.08 threshold, they fall within the acceptable boundary of < 0.10 for exploratory models analyzing complex behavioral constructs (e.g., Hair et al., 2010), though this warrants cautious interpretation (Figure 1).

**Figure 2:**
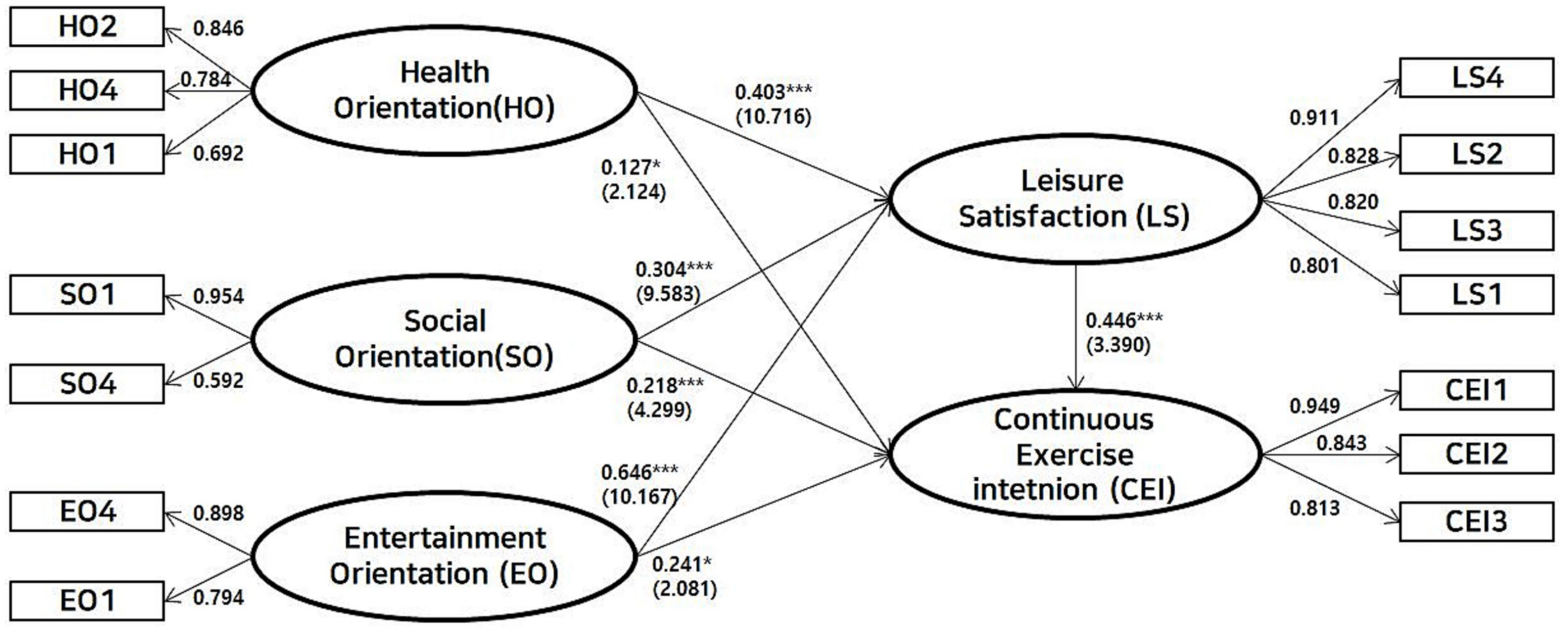
Result of the proposed research model.

The path analysis results are presented in Figure 2. In support of H1, all three motivation factors yielded a significant positive effect on leisure satisfaction. The strongest of these was entertainment orientation (*β* = 0.646, *p* < 0.001), followed by health orientation (*β* = 0.403, *p* < 0.001) and social orientation (*β* = 0.304, *p* < 0.001). Similarly, in support of H2, all three motivation factors were found to be significant positive predictors of continuous exercise intention. Again, entertainment orientation (*β* = 0.241, *p* < 0.05) and social orientation (*β* = 0.218, *p* < 0.001) were influential, along with health orientation (*β* = 0.127, *p* < 0.05). Finally, H3 was also strongly supported, as leisure satisfaction had a significant and positive effect on continuous exercise intention (*β* = 0.446, *p* < 0.001). And the regression analysis revealed that the independent variables (sports participation motivations) initially explained 38.0% of the variance ( $R^{2}$ = 0.380) in continuous exercise intention. With the introduction of the mediator variable, leisure satisfaction, the explanatory power of the model increased to 41.6% ( $R^{2}$ = 0.416). Furthermore, to rigorously verify the mediating effect of leisure satisfaction within this structural relationship, a hierarchical regression analysis applying a bootstrapping procedure with 5,000 resamples was conducted. The results demonstrated that the unstandardized coefficient of entertainment orientation on continuous exercise intention decreased from *B* = 0.555 (95% CI [0.392, 0.717], *p* < 0.001) to *B* = 0.374 (95% CI [0.143, 0.603], *p* = 0.002) upon the inclusion of leisure satisfaction. Concurrently, leisure satisfaction exhibited a significant positive effect on continuous exercise intention (*B* = 0.255, 95% CI [0.081, 0.455], *p* = 0.008). Based on these statistical indices, it can be interpreted that leisure satisfaction acts as a partial mediator in the relationship between entertainment orientation and continuous exercise intention. Taken together, these results confirm the proposed model. Crucially, while all tested motivational paths were significant, entertainment orientation emerged as the single most powerful predictor for both leisure satisfaction and, subsequently, continuance intention, highlighting its central role in the leisure experiences of adult Taekwondo practitioners.

## 4 Discussion

This study investigated the motivational structure of adult Taekwondo practitioners to understand how a traditional martial art is being redefined within the contemporary leisure landscape. The findings, particularly the primacy of entertainment motivation, offer significant contributions to leisure theory, challenging conventional distinctions between serious and casual leisure and highlighting the pervasive influence of the experience economy.

### 4.1 Theoretical implications

The main finding of this study is that entertainment orientation was the most powerful predictor of both leisure satisfaction and continuance intention. This result provides a compelling empirical challenge to the traditional understanding of activities like Taekwondo within the Serious Leisure Perspective (SLP). While martial arts are archetypal examples of ‘serious leisure’—requiring discipline, effort, and long-term commitment (Stebbins, 1992)—our findings suggest that participants are increasingly driven by motivations typically associated with ‘casual leisure’, namely immediate enjoyment and fun (Stebbins, 1997). This indicates a blurring of the lines between these two concepts. It suggests that for traditional activities to remain relevant, they must successfully integrate the hedonic rewards of casual leisure into the structured framework of serious leisure. Our study empirically demonstrates that the ‘serious’ pursuit of a skill is no longer sufficient; it must also be experientially rewarding and entertaining.

Furthermore, this finding strongly supports the application of the Experience Economy framework to participation-based leisure (Pine and Gilmore, 1999). The results imply that adult participants view their Taekwondo training not merely as an instructional service to be consumed, but as a holistic experience to be enjoyed. The fact that entertainment motivation—a factor directly tied to the quality of the immediate experience—dwarfs other motivations like health or social connection, suggests that the perceived value lies in the staging of the activity itself. This provides a new lens for leisure research, shifting the analytical focus from the outcomes of participation (e.g., health benefits) to the affective quality of the engagement itself.

A secondary but important finding was the non-significance of self-development and family orientations as motivators for this demographic. This suggests a departure from traditional narratives surrounding martial arts, which often emphasize character-building and discipline. For modern adult participants, it appears the immediate, personal leisure experience takes precedence over abstract, long-term goals or external social pressures.

Perhaps the most critical finding of this study was not only the primacy of entertainment but the statistical non-significance of the ‘self-development’ and ‘family’ orientation factors. In a traditional context, these motivations are foundational to martial arts practice, emphasizing character-building, discipline, and community (Ahn et al., 2009). The fact that they were statistically non-significant for this cohort—composed almost entirely of young male students—is a profound indicator of a cultural shift. Rather than indicating a definitive ‘value erosion’, the non-significance of traditional factors may be interpreted as a reweighting of the motivational structure observed specifically within this sample, which is predominantly composed of young male students. This cohort does not appear to be seeking a traditional, ‘serious’ leisure pursuit defined by discipline; they are seeking a contemporary, ‘experiential’ leisure activity that happens to be Taekwondo. This finding, rather than the model itself, offers the most significant contribution to the tension between SLP and the Experience Economy.

### 4.2 Practical implications

While this study’s focus is theoretical, the findings offer insights for practice. The primacy of entertainment suggests that practitioners and organisations involved in traditional physical activities should consider a strategic shift. Rather than marketing solely based on tradition, discipline, or health benefits, they should focus on designing and promoting the experiential quality of the activity. This does not mean abandoning the core principles of the practice, but rather enriching the participant experience by incorporating elements of fun, creativity, and cultural engagement. For example, integrating music, developing themed sessions, or creating more social and less competitive formats—perhaps shifting focus away from intense, competition-oriented sparring (Kyorugi) toward more cooperative or entertainment-based drills—can enhance the entertainment value without compromising the integrity of the martial art.

### 4.3 Limitations and future research

This study has several limitations that open avenues for future research. The sample was heavily skewed toward young, male students in Korea, which limits the generalisability of the findings. Future research should replicate this study with more diverse samples, including different age groups, genders, and cultural contexts, to explore whether the primacy of entertainment motivation is a global phenomenon. Furthermore, while our quantitative approach identified key relationships, qualitative methods such as in-depth interviews could provide a richer understanding of the lived experience of adult practitioners. Such research could explore how participants navigate the tensions between the ‘serious’ and ‘casual’ aspects of their Taekwondo experience, offering deeper insights into the evolving nature of modern leisure.

## 5 Conclusion

This study contributes to the leisure studies field by empirically demonstrating that, for a key demographic of young, male practitioners in South Korea, the modern practice of a traditional martial art is strongly driven by the pursuit of entertainment. This, combined with the notable absence of traditional motivators like self-development, suggests that in the contemporary leisure market, the boundaries between serious and casual leisure are becoming increasingly porous, and the principles of the experience economy are highly relevant to understanding participant behaviour. To remain a compelling leisure choice for this specific demographic of young adult practitioners, it is suggested that traditional activities like Taekwondo be managed not merely as a path to skill and discipline, but also as a source of enjoyable and memorable experiences.

## Data Availability

The raw data supporting the conclusions of this article will be made available by the authors, without undue reservation.
